# Blood Group Antigen Combinations and COVID-19: Complexity, Associations and Possible Clinical Relevance

**DOI:** 10.3390/life16061038

**Published:** 2026-06-22

**Authors:** Jolanta Wrobel, Ewa Jablonska, Krzysztof Matuk, Agnieszka Zebrowska, Piotr Radziwon, Wioletta Ratajczak-Wrona

**Affiliations:** 1 Regional Centre for Transfusion Medicine, 15-950 Bialystok, Poland; jwrobel@rckik.bialystok.pl (J.W.);; 2 Department of Immunology, Medical University of Bialystok, ul. J. Waszyngtona 15A, 15-269 Bialystok, Poland; 3 Imago Sp. z o.o., ul. Przedzalniana 6L lok. 14, 15-688 Bialystok, Poland; 4 Department of Haematology, Medical University of Bialystok, ul. M. Skłodowskiej-Curie 24A, 15-276 Bialystok, Poland

**Keywords:** group antigens, COVID-19

## Abstract

Background: The aim of the study was to investigate whether red blood cell antigens (A, B, D, C^w^, C, c, E, e, K, k, Jk^a^, Jk^b^, Fy^a^, Fy^b^, Le^a^, Le^b^, M, N, S, s, and P1) from clinically relevant blood group systems were associated with susceptibility to COVID-19. Methods: This exploratory case-control study was carried out on a group of 263 donors from the Regional Center for Transfusion Medicine, Bialystok, Poland (including 121 convalescents and 142 healthy subjects). The microplate method was applied to examine the expression of 21 antigens from eight clinically relevant systems in the donors’ red blood cells. Results: No significant correlation was found between any single blood group and susceptibility to COVID-19. For a more detailed analysis, we adopted an approach involving 3-, 4- and 5-element mutual combinations of antigens. In this exploratory analysis of multi-antigen combinations, nominal statistical significance was found for a number of associations, but none remained statistically significant after adjustment for multiple testing. In the case of three-antigen associations, the strongest association was observed for cc combined with Ee and kk, with the effect size showing approximately 4× higher odds of COVID-19 (OR = 4.49, *p* = 0.020); in the case of four-antigen combinations, the strongest association was found for RhD(+) combined with kk, Fy^b^ and P1(−), as well as RhD(+) combined with cc, Ee and kk, also indicating 4× higher odds of COVID-19 (OR = 4.49 *p* = 0.016). For five-antigen combinations, the strongest association was observed for blood type O combined with kk, Le^b^, P1(+) and MM, with the association strength reaching an OR = 4.44, *p* = 0.025. Conclusions: The findings suggest that analyses based solely on single blood group antigens may have limited value for assessing the susceptibility to COVID-19. In contrast, combinations of red blood cell antigens may provide more reliable correlations with the susceptibility to COVID-19. However, these findings should be interpreted with caution and require confirmation on larger and more representative populations.

## 1. Introduction

To date, 48 distinct blood group systems and hundreds of individual blood antigens have been recognized and classified. Most blood group antigens are also found in cells other than erythrocytes. Group antigens are incorporated into proteins, glycoproteins or glycosphingolipids. Their synthesis is controlled by genes encoding integral proteins, surface proteins or glycosyltransferases [1,2].

Antigens can play a number of roles, e.g., as transporters, adhesion molecules, enzymes, structural proteins and receptors for proteins. However, erythrocyte antigens are also responsible for hemolytic post-transfusion reactions. Clinically important group systems include, among others, ABO, Rh, Kell, MNS, Kidd, Duffy, P1PK, and Lewis [2,3,4,5].

In recent years, establishing associations between blood groups and various diseases has been the focus of considerable research efforts [6]. The studies have focused on the ABO system [7,8,9]. An increased incidence of gastric cancer has been observed in people with blood group A [10,11]. Certain pathogens may contain substances that are analogous to human antigens; as a result, the immune systems of people with the corresponding ABO blood group fail to produce an adequate response against them, thus increasing the risk of death (plague bacterium, smallpox virus) [2,7].

Studies have reported that RhD-positive individuals are protected from the effects of latent toxoplasmosis caused by the eukaryotic parasite Toxoplasma gondii [12,13]. However, antigens of the MNS system function as receptors for a variety of bacteria and parasites, making them primarily responsible for certain infections, including the infection causing the most severe form of malaria—Plasmodium falciparum [14]. The sialylated forms of Lea and Leb can be ligands for, among others, selectins and bacteria [7,15].

Correlation with viral resistance outside the ABO system has also been demonstrated for the Duffy, P1PK, and Lewis systems. The Duffy antigens found on erythrocytes have been shown to bind HIV-1 virions in the blood, and thus may act as a reservoir for the virus prior to the transmission to leukocytes [16,17]. Depending on the presence of P1PK system antigens on leukocytes, cells may be either susceptible or resistant to HIV infection, or may provide a receptor for, among others, human parvovirus B19 [15]. In addition, in individuals with Lewis antigens, the binding to norovirus (NoVs)—the main causative agent of acute nonbacterial gastroenteritis in humans—is stronger [18].

Attempts have also been made to establish the correlation between blood groups and severe respiratory distress syndrome—such as that caused by COVID-19 [19,20,21]. Patients with COVID-19 often have high levels of cytokines, reduced lymphocyte levels, coagulation abnormalities and vascular damage. Therefore, affordable rapid screening tests that assess biomarkers (including the C-reactive protein, ferritin, D-dimer, and IL-6) are being researched to evaluate disease severity and potential complications. This strategy can potentially focus on assaying antigens from the major ABO and Rh group systems, as those assays are rapid and inexpensive. Furthermore, in some countries, the relevant data are readily available in patient records [20,22,23,24].

To date, many researchers have been focused chiefly on establishing the correlation between the two group systems (ABO and Rh) and the COVID-19 disease. However, only a number of researchers have investigated the frequency of infections and other blood cell antigens (including Lewis and T) in those patients [9,25,26,27,28].

In view of the data presented above, the research objective was to test the following hypotheses: (1) there is a correlation between red blood cell antigens (from clinically relevant group systems) and a predisposition to COVID-19; (2) specific combinations of red blood cell antigens are associated with an increased susceptibility to COVID-19.

## 2. Materials and Methods

### 2.1. Study Participants

This study was conducted between 1 May and 30 July 2021 in the Regional Centre for Transfusion Medicine in Bialystok, Poland.

The convalescent group consisted of 121 voluntary blood donors (11 women and 110 men) from the Regional Centre for Transfusion Medicine in Bialystok, Poland, in whom the SARS-CoV-2 virus was previously detected using a PCR or antigen test. The donors were recruited after a detailed interview and a standard physical examination. Their history of SARS-CoV-2 infection was confirmed based on the source documentation provided. None of the donors required hospitalization during COVID-19, and no severe disease cases were reported. The participants had no known significant comorbidities at the time of blood donation. All donors were of White/Caucasian ethnicity and originated from the Podlaskie Voivodeship in northeastern Poland. The age of the convalescent donors ranged from 20 to 65 years. All donors received information about the study plan and methods, and gave written consent to participate in it. The study was approved by the Ethics Committee of the Medical University of Bialystok (No. APK.002.17.2021).

The control group consisted of 142 voluntary blood donors (38 women and 104 men) from the Regional Centre for Transfusion Medicine in Bialystok, in whom the presence of the SARS-CoV-2 virus had not been previously detected by a PCR or antigen test. The donors were recruited after a detailed interview and a standard physical examination. Their age ranged from 20 to 65 years. All donors received information about the study plan and methods, and gave written consent to participate in it. The study was approved by the Ethics Committee of the Medical University of Bialystok (No. APK.002.17.2021).

After obtaining the consent of honorary blood donors, 2 mL of venous blood was collected from the antecubital vein into anticoagulant-containing tubes. In the collected samples, antigens on the red blood cells were identified (A, B, D, C^w^, C, c, E, e, K, k, Jk^a^, Jk^b^, Fy^a^, Fy^b^, Le^a^, Le^b^, M, N, S, s, and P1). All methods were performed in accordance with the relevant guidelines and regulations.

### 2.2. Reagents

Anti-A IgM clone: Birma-1, anti-B IgM clone: LB-2, anti-D IgM clone: RUM-1, anti-D IgG + IgM clone: TH-28/MS-26, anti-Cw IgM clone: MS-110, anti-C IgM clone: MS-24, anti-c IgM clone: MS-33, anti-E IgM clone: MS-80/MS-258, anti-e IgM clone: MS-16/MS-21/MS-63 and anti-K IgM clone: MS-56 were purchased from MILLIPORE (Billerica, MA, USA). Bromeline PK was purchased from DIAGAST (Loos, France). Anti-Jk^a^ IgM gamma-clone, anti-Jk^b^ IgM gamma-clone, anti-P1 IgM clone: P3NIL100, anti-M IgM clone: M-11H2, anti-N IgM clone: 1422-C7, anti-k micro IgG human polyclonal, anti-Fy^a^ micro IgG human polyclonal, anti-Fy^b^ micro IgG human polyclonal, anti-S micro IgG human polyclonal, anti-s micro IgG human polyclonal, Capture^®^ LISS, anti-Lea IgM gamma-clone, anti-Leb IgM gamma-clone and indicator cells (Capture-R^®^ Ready Indicator Red Cells) were purchased from IMMUCOR (Norcross, GA, USA). ID-diluent 2 was purchased from BioRad Laboratories (Hercules, CA, USA). Bromeline PK was purchased from DIAGAST (Loos, France). ABO system standard cells were produced at the Regional Centre for Transfusion Medicine.

### 2.3. Rbc Antigen Expression

All tests were conducted between 1 May and 30 July 2021 in the Donor Blood Groups Laboratory of the Regional Centre for Transfusion Medicine, Bialystok, Poland. Nine red blood cell antigens (A, B, D, C^w^, C, c, E, e, and K) were identified using the microplate technique on a PK7300 analyzer from BECKMAN COULTER (Brea, CA, USA). Ten antigens (k, Jk^a^, Jk^b^, Fy^a^, Fy^b^, M, N, S, s, and P1) were identified using the microplate method on a NEO IRIS analyzer from IMMUCOR (Norcross, GA, USA). Since Le^a^ and Le^b^ antigens cannot be identified using these instruments, they were detected using the manual tube method.

### 2.4. Identification of Antigens from the ABO System and the Rh System, and K Antigen from the Kell System, Using the Microplate Technique

Using the Bromeline PK enzyme (DIAGAST, Loos, France), the corresponding antigen present on the donor cells was combined with IgM monoclonal antibodies in the reagent. Agglutination occurred in microcells on a microplate, indicating the presence of the antigen in question on the test cells; the lack of agglutination indicated the absence of the antigen. In the PK7300 analyzer, appropriate reagents containing monoclonal antibodies of the IgM class were placed in microplates with diluted test cells in 0.9% NaCl. The microplates were incubated at 28 °C for 1 h, followed by shaking. Reaction readings were recorded in the database of the PK7300 Automated Microplate System (Beckman Coulter, Brea, CA, USA). The operator checked for and validated agglutination or the lack thereof.

### 2.5. Identification of Antigens from the Kidd System, P1 Antigen from the P1PK System, and M and N Antigens from the MNS System Using the Microplate Technique

IgM monoclonal antibodies were used to identify the corresponding antigen on the test cells obtained from donors. Reactions were carried out in microcells on a microplate. Agglutination indicated that the given antigen was present on the test cells, while the lack of agglutination indicated the absence of the antigen. In the analyzer, appropriate reagents containing monoclonal antibodies of the IgM class were added to microplates with diluted test cells. The microplates were incubated at 23 °C for 10 min, centrifuged, read and stored in the NEO Iris^®^ analyzer (Immucor, Norcross, GA, USA) database. The operator checked for and validated agglutination or the lack thereof.

### 2.6. Identification of Antigens from the Duffy System, k Antigen from the Kell System, and S and s Antigens from the MNS System Using the Microplate Technique

Capture-R^®^ Select (IMMUCOR) microplates were used to detect group antigens using human IgG polyclonal reagents. In the analyzer, a suspension of red blood cells of the test cells in the Capture^®^ LISS reagent (IMMUCOR) was added to the microplates. The microplates with the donor test cells were incubated for 20 min at 39 °C, during which the test cells were bound to the surface of the microplates. Next, appropriate reagents containing human IgG class antibodies were added; the antibodies bound to the corresponding antigen on the red cells attached to the bottom of the microcell. The microplates were again subjected to a short incubation at 39 °C. After the incubation, unbound immunoglobulins were washed out of the microplates, to which Capture-R^®^ Ready Indicator Red Cells (a suspension of red cells coated with anti-IgG antibodies; IMMUCOR) were added. The microplates were then centrifuged, during which the Capture-R^®^ Ready Indicator Red Cells (IMMUCOR) contacted the reagent’s IgG antibodies. In the agglutination reaction, the antibodies bound to the donor test cells and the antibodies coating Capture-R^®^ Ready Indicator Red Cells (by IMMUCOR) were formed. In the absence of an antigen–antibody reaction (negative result), the indicator cells were not blocked during centrifugation and accumulated at the bottom of the wells, forming tightly compacted pellets. Reaction readings were recorded in the NEO IRIS analyzer database. The operator checked for and validated agglutination or the lack thereof.

### 2.7. Identification of Lea and Leb Antigens from the Lewis System Using the Manual Tube Method

Donor cells were washed with 0.9% NaCl and then used to prepare a 4% suspension. Le^a^ and Le^b^ antigens were identified according to the manufacturer’s instructions using the prepared 4% suspension of donor red blood cells and anti-Le^a^ and anti-Le^b^ monoclonal antibodies. Agglutination (on a scale of 1+ to 4+) indicated the presence of the tested antigen on the test cells, whereas the lack of agglutination indicated the absence of the antigen.

### 2.8. Statistical Analysis

Statistical analyses were performed using R: A Language and Environment for Statistical Computing, version 4.4.2. This study was exploratory; therefore, no formal sample size calculations were performed or confirmatory hypothesis testing framework applied. In the summary of characteristics, age was presented as the mean ± standard deviation (given its normal distribution). Normality was evaluated using the Shapiro–Wilk test, with additional verification through skewness and kurtosis. Variance homogeneity was checked using Levene’s test. The characteristics of the study groups were compared with Student’s *t*-test, Pearson’s chi-square test or Fisher’s exact test, as appropriate. The analysis followed a stepwise workflow comprising screening, modeling, and validation stages. All unique combinations of 3, 4, and 5 antigens were identified in the study population, and only those found in at least 5 participants in each study group were retained for further analysis (n = 1139, n = 2092, and n = 1889 combinations for 3-, 4-, and 5-antigen sets, respectively). The incidence of these combinations was statistically compared between convalescents and the control group with Pearson’s chi-square test. Combinations demonstrating nominally significant inter-group differences (*p* < 0.05) were selected for further exploratory analyses (n = 25 for 3-antigen, n = 31 for 4-antigen, and n = 25 for 5-antigen combinations). Logistic regression was applied to estimate the strength and direction of the correlations between individual antigen combinations and prior COVID-19 infections. Logistic regression models were adjusted for gender and age as potential confounders. Odds ratios (ORs) with 95% confidence intervals were applied to quantify the strength and direction of association. Internal validation of logistic regression models was performed using bootstrap resampling (500 replicates), with percentile-based confidence intervals for odds ratios derived from bootstrap distributions to assess the stability of the estimates.

A false discovery rate (FDR) correction was primarily applied within each antigen-size family for all comparisons of all evaluated combinations. As a sensitivity analysis, FDR-adjusted *p*-values were also calculated for the logistic regression models of selected combinations.

A receiver operating characteristic (ROC) analysis was conducted to evaluate the discriminatory performance of antigen combinations for COVID-19 incidence. All variables were complete, and no missing-data-handling procedures were required. A two-sided *p*-value <0.05 was considered nominally statistically significant.

## 3. Results

### 3.1. Characteristics of Entire Study Group and Study Subgroups

A detailed overview of the frequency of occurrence of the tested antigens in the study groups (convalescents and healthy persons) is shown in Table 1. The groups differed by sex and age, with a lower proportion of male patients (90.0% vs. 73.2%, *p* < 0.001) and a higher age (38.38 ± 9.34 vs. 34.02 ± 9.51, *p* < 0.001) observed among convalescents compared to the control group.

### 3.2. Exploratory Screening of Antigen Combinations

A total of 1139 three-element, 2092 four-element and 1889 five-element antigen combinations meeting the predefined frequency criteria were evaluated in the exploratory screening analysis. Based on the nominally significant inter-group differences (Pearson’s chi-square test, *p* < 0.05), 25 three-element, 31 four-element and 25 five-element antigen combinations were selected (Supplementary Tables S4, S8 and S12) as candidate combinations for subsequent logistic regression analyses. However, after an FDR correction was applied to all the evaluated combinations within each antigen-size family, no correlation remained statistically significant. Therefore, the findings presented below should be interpreted as exploratory and hypothesis-generating.

### 3.3. Correlation Between Antigen Combinations and COVID-19 Incidence—Combinations of Three Antigens

All of the three-element antigen combinations selected based on the nominal inter-group differences in the exploratory screening are presented in Table 2. Six combinations were found to have been related to increased odds of COVID-19 morbidity, with the effect size ranging from 2 to 4, depending on the combination.

The strongest effects were observed for cc combined with Ee and kk, as well as cc combined with Ee and RhD(+), which were associated with 4-fold higher odds (OR = 4.49 CI95 [1.69;13.36], *p* = 0.020, and OR = 4.15 CI95 [1.64;11.63], *p* = 0.020, respectively).

The odds of COVID-19 incidence were approximately 3-fold higher in the case of kk combined with P1(−) and Cc, as well as kk combined with P1(−) and RhD(+) (OR = 2.60 CI95 [1.20;5.91], *p* = 0.029, and OR = 2.63 CI95 [1.44;4.91], *p* = 0.016, respectively).

The odds of COVID-19 incidence were approximately 2-fold higher in the case of Le^b^ combined with P1(−) and kk, as well as Le^b^ combined with P1(−) and RhD(+) (OR = 1.98 CI95 [1.10;3.58], *p* = 0.032, and OR = 2.03 CI95 [1.11;3.79], *p* = 0.032, respectively).

Bootstrap internal validation indicated that the direction and magnitude of the observed correlations were generally consistent across resamples (Supplementary Table S5).

The receiver operating characteristic (ROC) analysis indicated a low discriminating ability of the antigen combinations for COVID-19 incidence (AUC < 0.6, Supplementary Table S6).

### 3.4. Correlations Between Antigen Combinations and COVID-19 Incidence—Combinations of Four Antigens

All of the four-element antigen combinations selected on the basis of the nominal inter-group differences in the exploratory screening are presented in Table 3. It was found that four combinations were correlated with increased odds of COVID-19 morbidity, with the effect size ranging from 2 to 4, depending on the combination.

The strongest correlations with COVID-19 incidence were observed for RhD(+) combined with kk, Fyb and P1(−) and RhD(+) combined with kk, cc and Ee, which increased the odds 4-fold (OR = 4.49 CI95 [1.61;14.33], *p* = 0.016, and OR = 4.49 CI95 [1.69;13.36], *p* = 0.016, respectively).

Also, 2-fold higher odds were noted in the case of RhD(+) combined with kk, P1(−) and Cc as well as RhD(+) combined with kk, P1(−) and Le^b^ (OR = 2.49 CI95 [1.14;5.70], *p* = 0.033, and OR = 2.29 CI95 [1.22;4.38], *p* = 0.019, respectively).

Bootstrap internal validation indicated that the direction and magnitude of the observed associations were generally consistent across resamples (Supplementary Table S9).

The receiver operating characteristic (ROC) analysis indicated a low discriminating ability of the antigen combinations for COVID-19 incidence (AUC < 0.6, Supplementary Table S10).

### 3.5. Correlations Between Antigen Combinations and COVID-19 Incidence—Combinations of Five Antigens

All of the five-element antigen combinations selected on the basis of the nominal inter-group differences in the exploratory screening are presented in Table 4. One combination was correlated with increased odds of COVID-19 morbidity: blood type O combined with kk, Leb, P1(+) and MM, which was associated with 4-fold higher odds, OR = 4.44 CI95 [1.51;15.39], *p* = 0.025. Bootstrap internal validation indicated that the direction and magnitude of the observed correlation was consistent across resamples (Supplementary Table S13).

The receiver operating characteristic (ROC) analysis indicated a low discriminating ability of the antigen combination for COVID-19 incidence (AUC = 0.536, Supplementary Table S14).

### 3.6. Correlations Between Antigen Combinations and Undocumented COVID-19 Incidence—Combinations of Three, Four or Five Antigens

Among the assessed three-element antigen combinations, blood type A combined with Cc and P1(+) as well as ee combined with ss and P1(+) were associated with the lowest odds of a prior COVID-19 infection: OR = 0.29 CI95 [0.11;066], *p* = 0.020, and OR = 0.29 CI95 [0.14;056], *p* = 0.009, respectively (Table 2). Out of all four antigenic combinations, the lowest odds were found for the combination of ee, Le^b^, ss and P1(+), resulting in an OR = 0.21 CI95 [0.09;0.45], *p* = 0.003 (Table 3). Out of all five antigenic combinations, the lowest odds of COVID-19 infection were found in the case of the combination of blood ee, Jk^a^Jk^b^, Le^b^, ss and P1(+), with an OR = 0.19 CI95 [0.06;0.50], *p* = 0.017 (Table 4). Bootstrap internal validation indicated that the direction and magnitude of the observed correlations were generally consistent across resamples (Supplementary Tables S5, S9 and S13).

The receiver operating characteristic (ROC) analysis indicated a low discriminating ability of the combination of these antigens (AUC < 0.6, Supplementary Tables S7, S11 and S15).

## 4. Discussion

To date, attempts to establish the correlation between blood groups and the COVID-19 disease have primarily been focused on the phenotype of one or two systems [29,30]. A study by Li et al. conducted on approximately 2000 COVID-19 patients found a correlation between blood group ABO and susceptibility to COVID-19 [31]. The authors reported that people with blood group A may be at an increased risk of SARS-CoV-2 infection and severe COVID-19, whereas those with blood group O are at a significantly lower risk. Most studies to date have focused primarily on the search for specific blood groups or antigens that could be responsible for the increased risk of COVID-19. Unlike previous studies, our approach included mutual combinations of antigens; namely, we created three-, four- and five-element combinations. In the subsequent stages of the analysis, we took into account only the combinations that occurred at least five times in each of the study groups. After performing a statistical comparison between the study groups in terms of the occurrence of each combination, for further analysis, we selected only those combinations for which a significant difference between the groups was confirmed. A logistic regression analysis showed that there was a 4-fold increase in the risk of COVID-19 for the following combination of antigens: cc combined with Ee and kk; RhD(+) combined with cc and Ee; RhD(+) combined with kk, Fy^b^ and P1(−); RhD(+) combined with cc, Ee and kk; and blood type O combined with kk, Le^b^, P1(+) and MM. The observation regarding the absence of the P1 antigen is particularly puzzling in light of the data, where, depending on the presence of P1PK system antigens on leukocytes, cells can be either susceptible or resistant to HIV infection, or provide a receptor for, among others, parvovirus B19 [15].

In transfusion serology, homozygous cells, which have the antigen in a double dose, play an important role, making it possible to detect antibodies with low titers. This observation became the starting point for a different question: can homozygosity for specific red blood cell antigens contribute to protection against a SARS-CoV-2 infection? An analysis of three-, four-, and five-element combinations showed that the presence of Leb, M, and S antigens in double doses, in reciprocal combinations with blood group A, significantly increased the probability of protection against COVID-19. This observation becomes particularly significant in the context of the data on antigen A, as it is known that the number of its determinants on the erythrocyte is about 1 million in blood group A1. At the same time, GYPA is the most abundant sialoglycoprotein on the surface of erythrocytes. In addition, GYPA/B are found exclusively on the surfaces of erythrocytes. There are data available that show that pathogens [including hepatitis A virus and influenza types A and B] use erythrocyte glycoproteins as receptors to invade target cells [32,33]. Interestingly, erythrocyte surface glycoproteins have been proposed to participate in host–pathogen interactions by binding certain microorganisms [34,35]. Although the relevance of this mechanism to SARS-CoV-2 infection remains unclear, the variability in red blood cell antigen expression may contribute to differences in susceptibility to viral infections.

Since the A, Le^b^, M, and S antigens originate from three different systems, the question arose about the potential relationship between those antigens. The Lewis phenotype may be modified by the ABO phenotype: A1 individuals have a lower expression of Lewis antigens than O individuals [2,36]. In addition, another common feature of ABO and Lewis antigens is that they are formed by the action of their respective glycotransferases. Furthermore, glycophorins A and B are believed to form a protective envelope around erythrocytes due to their numerous sugar chains. M and N antigens differ at positions 1 and 5 of the amino acid chain of glycophorin A: M antigen: Ser-Ser-Thr-Thr-Gly; N antigen: Leu-Ser-Thr-Thr-Glu. Natural antibodies from the ABO system may provide up to 40% protection against viral transmission between ABO-mismatched individuals [31,37].

Pendu et al. reported that the proteins of viruses, including SARS-CoV-2, are richly glycosylated and, like viruses, may be coated with allogeneic carbohydrate epitopes of ABO blood groups [38]. Therefore, glycosylation may be used by enveloped viruses as a protective mechanism and perhaps even as a “Trojan horse” [39,40]. In addition, ABO-system antigens can affect carbohydrate–carbohydrate (CCI) binding, which can induce the attachment of the viral spike protein to the host cell receptor [15,40]. Different results regarding antigens from the Lewis system were obtained by Matzhold et al. [10]. They suggest that the Le(a−b−) phenotype is a protective factor against severe disease, while the Le(a+b−) and Le(a−b+) phenotypes may play a role in the progression of the disease.

This study has a number of limitations. First, a large number of antigen combinations were evaluated, increasing the risk of false-positive findings. After applying the FDR correction to the initial comparisons stage, none of the observed correlations remained statistically significant; therefore, the results should be considered exploratory and hypothesis-generating. Second, some antigen combinations occurred infrequently, resulting in wide confidence intervals and limited precision of the selected estimates. Further, some higher-order antigen combinations represented extensions of lower-order combinations and thus produced similar frequencies and effect estimates. In addition, the study groups differed in terms of sex composition, although adjusted analyses accounted for sex as a potential confounder. Finally, although internal bootstrap validation demonstrated generally consistent effect estimates across resamples, external validation in independent cohorts is required.

Another limitation of the present study was the relatively small number of donors, although it remained within the acceptable range for statistical analyses. Furthermore, the study population consisted exclusively of voluntary blood donors, i.e., a select and generally healthier population that may not fully reflect the characteristics of the general population. The study was also limited by the lack of epidemiological data, including vaccination status and information regarding the occupational or environmental exposure risk. An additional limitation concerns the control group, which was established based on medical history and interview data only; therefore, asymptomatic or previously unrecognized SARS-CoV-2 infections among control participants cannot be excluded. Despite the use of rigorous statistical methods designed to reduce the risk of overinterpretation, further studies are needed to confirm the importance of specific combinations as predictive factors in viral disease incidence.

## 5. Conclusions

The results of our study clearly indicate that considering single antigens in terms of predisposition to COVID-19 morbidity is subject to high error margins. In contrast, combinations of red blood cell antigens may provide more reliable correlations with susceptibility to COVID-19.

## Figures and Tables

**Table 1:** Study group characteristics.

Variable	Total Group, n (%) (n = 263)	Convalescents, n (%) (n = 121)	Control Group, n (%) (n = 142)	*p*
Gender, male; n (%)	214 (81.4)	110 (90.9)	104 (73.2)	**<0.001**
Age, years, mean ± SD	36.03 ± 9.66	38.38 ± 9.34	34.02 ± 9.51	**<0.001**
Blood type; n (%)				
A	119 (45.2)	49 (40.5)	70 (49.3)	0.061
AB	8 (3.0)	7 (5.8)	1 (0.7)	
B	44 (16.7)	19 (15.7)	25 (17.6)	
O	92 (35.0)	46 (38.0)	46 (32.4)	
RhD; n (%)				
Presence of D antigen—RhD(+)	217 (82.5)	103 (85.1)	114 (80.3)	0.386
No D antigen—RhD(−)	46 (17.5)	18 (14.9)	28 (19.7)	
Antigen phenotype: C, C^w^, c; n (%)				
CC	46 (17.5)	20 (16.5)	26 (18.3)	0.721
Cc	113 (43.0)	55 (45.5)	58 (40.8)	
cc	85 (32.3)	40 (33.1)	45 (31.7)	
CC^w^	6 (2.3)	2 (1.7)	4 (2.8)	
C^w^c	13 (4.9)	4 (3.3)	9 (6.3)	
Antigen phenotype: E, e; n (%)				
EE	7 (2.7)	2 (1.7)	5 (3.5)	0.131
ee	202 (76.8)	88 (72.7)	114 (80.3)	
Ee	54 (20.5)	31 (25.6)	23 (16.2)	
Occurrence of Kell system phenotypes; n (%)				
K [K+k−]	1 (0.4)	0 (0.0)	1 (0.7)	0.302
k [K−k+]	246 (93.5)	116 (95.9)	130 (91.5)	
Kk [K+k]	16 (6.1)	5 (4.1)	11 (7.7)	
Occurrence of Duffy system phenotypes; n (%)				
Fy^a^ [Fy(a+b−)]	52 (19.8)	23 (19.0)	29 (20.4)	0.948
Fy^b^ [Fy(a−b+)]	83 (31.6)	38 (31.4)	45 (31.7)	
Fy^a^Fy^b^ [Fy(a+b+)]	128 (48.7)	60 (49.6)	68 (47.9)	
Occurrence of Kidd system phenotypes; n (%)				
Jk^a^ [Jk(a+b−)]	58 (22.1)	30 (24.8)	28 (19.7)	0.532
Jk^b^ [Jk(a−b−)]	57 (21.7)	27 (22.3)	30 (21.1)	
Jk^a^Jk^b^ [Jk(a+b+)]	148 (56.3)	64 (52.9)	84 (59.2)	
Occurrence of Lewis system phenotypes; n (%)				
Le^a^ [Le(a+b−)]	27 (10.3)	12 (9.9)	15 (10.6)	0.815
Le^b^ [Le(a−b+)]	211 (80.2)	96 (79.3)	115 (81.0)	
Absence of Le^a^ and absence of Le^b^ [Le(a−b−)]	25 (9.5)	13 (10.7)	12 (8.5)	
Occurrence of P1PK system phenotypes; n (%)				
P1(+) [P1]	177 (67.3)	73 (60.3)	104 (73.2)	**0.036**
P1(−) [P2]	86 (32.7)	48 (39.7)	38 (26.8)	
Occurrence of MNS system phenotypes:				
Antigen phenotype: M, N; n (%)				
MM	114 (43.3)	51 (42.1)	63 (44.4)	0.576
NN	29 (11.0)	16 (13.2)	13 (9.2)	
MN	120 (45.6)	54 (44.6)	66 (46.5)	
Antigen phenotype: S, s; n (%)				
SS	36 (13.7)	15 (12.4)	21 (14.8)	0.183
ss	104 (39.5)	42 (34.7)	62 (43.7)	
Ss	123 (46.8)	64 (52.9)	59 (41.5)	

Comparison performed with Student’s *t*-test (age) and Pearson’s chi-square test or Fisher’s exact test (categorical variables, as appropriate).

**Table 2:** Exploratory nominal correlations of 3-antigen combinations with COVID-19 incidence: comparison between convalescents and controls and adjusted logistic regression analysis.

Combination of Blood Group/Antigens	Convalescents, n (%) (n = 121)	Control Group, n (%) (n = 142)	Adjusted Logistic Regression (Gender and Age)		
			OR	95% CI	*p* *
Combinations positively correlated with increased odds of COVID-19 incidence (OR > 1)					
cc|Ee|kk	16 (13.2)	7 (4.9)	4.49	1.69–13.36	**0.020**
RhD(+)|cc|Ee	17 (14.0)	8 (5.6)	4.15	1.64–11.63	**0.020**
Cc|kk|P1(−)	22 (18.2)	12 (8.5)	2.60	1.20–5.91	**0.029**
kk|P1(−)|ss	21 (17.4)	12 (8.5)	1.94	0.90–4.34	0.098
RhD(+)|kk|P1(−)	40 (33.1)	26 (18.3)	2.63	1.44–4.91	**0.016**
kk|Le^b^|P1(−)	40 (33.1)	28 (19.7)	1.98	1.10–3.58	**0.032**
RhD(+)|Le^b^|P1(−)	35 (28.9)	25 (17.6)	2.03	1.11–3.79	**0.032**
Combinations negatively correlated with COVID-19 incidence (OR < 1)					
Blood type A|RhD(+)|kk	36 (29.8)	60 (42.3)	0.59	0.34–1.00	0.055
ee|kk|P1(+)	49 (40.5)	77 (54.2)	0.50	0.30–0.85	**0.025**
ee|Le^b^|P1(+)	41 (33.9)	68 (47.9)	0.52	0.30–0.88	**0.027**
ee|Jk^a^Jk^b^|P1(+)	25 (20.7)	46 (32.4)	0.55	0.30–1.00	0.055
ee|Le^b^|ss	26 (21.5)	48 (33.8)	0.47	0.26–0.85	**0.025**
Blood type A|Le^b^|P1(+)	22 (18.2)	42 (29.6)	0.50	0.27–0.92	**0.035**
ee|Jk^a^Jk^b^|ss	18 (14.9)	37 (26.1)	0.48	0.24–0.92	**0.035**
Blood type A|kk|P1(+)	25 (20.7)	49 (34.5)	0.47	0.26–0.84	**0.025**
Blood type A|RhD(+)|P1(+)	24 (19.8)	49 (34.5)	0.47	0.26–0.84	**0.025**
ee|Fy^a^Fy^b^|ss	12 (9.9)	28 (19.7)	0.42	0.19–0.89	**0.035**
kk|P1(+)|ss	19 (15.7)	43 (30.3)	0.42	0.22–0.79	**0.025**
Fy^a^Fy^b^|P1(+)|ss	9 (7.4)	24 (16.9)	0.42	0.17–0.97	0.055
Jk^a^Jk^b^|P1(+)|ss	11 (9.1)	30 (21.1)	0.39	0.17–0.84	**0.029**
Blood type A|Cc|P1(+)	8 (6.6)	25 (17.6)	0.29	0.11–0.66	**0.020**
Blood type A|P1(+)|ss	8 (6.6)	25 (17.6)	0.30	0.12–0.69	**0.025**
Le^b^|P1(+)|ss	15 (12.4)	43 (30.3)	0.32	0.16–0.62	**0.013**
ee|P1(+)|ss	15 (12.4)	44 (31.0)	0.29	0.14–0.56	**0.009**

OR—odds ratio, CI—confidence interval. *p* *—outcome of logistic regression analysis: each row represents outcome of one logistic regression model with COVID-19 incidence as the dependent variable, the given combination of antigens as the independent variable, and gender and age as covariates; the *p* values were adjusted with the FDR method across the analyzed logistic regression models.

**Table 3:** Exploratory nominal associations of 4-antigen combinations with COVID-19 incidence: comparison between convalescents and controls and adjusted logistic regression analysis.

Combination of Blood Group/Antigens	Convalescents, n (%) (n = 121)	Control Group, n (%) (n = 142)	Adjusted Logistic Regression (Gender and Age)		
			OR	95% CI	*p* *
Combinations positively correlated with increased odds of COVID-19 incidence (OR > 1)					
RhD(+)|kk|Fy^b^|P1(−)	15 (12.4)	6 (4.2)	4.49	1.61–14.33	**0.016**
RhD(+)|cc|Ee|kk	16 (13.2)	7 (4.9)	4.49	1.69–13.36	**0.016**
kk|Leb|P1(−)|ss	18 (14.9)	8 (5.6)	2.23	0.94–5.74	0.079
RhD(+)|kk|P1(−)|ss	18 (14.9)	9 (6.3)	2.21	0.95–5.49	0.077
RhD(+)|Cc|kk|P1(−)	21 (17.4)	12 (8.5)	2.49	1.14–5.70	**0.033**
RhD(+)|kk|Le^b^|P1(−)	34 (28.1)	22 (15.5)	2.29	1.22–4.38	**0.019**
Combinations negatively correlated with COVID-19 incidence (OR < 1)					
Blood type A|RhD(+)|kk|Jk^a^Jk^b^	19 (15.7)	38 (26.8)	0.54	0.28–1.01	0.064
ee|kk|Jk^a^Jk^b^|ss	17 (14.0)	35 (24.6)	0.49	0.24–0.94	**0.044**
Blood type A|kk|Le^b^|P1(+)	19 (15.7)	39 (27.5)	0.47	0.24–0.89	**0.033**
ee|kk|Jk^a^Jk^b^|P1(+)	22 (18.2)	44 (31.0)	0.50	0.27–0.92	**0.036**
Blood type A|RhD(+)|Le^b^|P1(+)	18 (14.9)	40 (28.2)	0.45	0.23–0.86	**0.027**
Blood type A|RhD(+)|kk|P1(+)	20 (16.5)	45 (31.7)	0.42	0.22–0.78	**0.016**
RhD(+)|Le^b^|P1(+)|ss	12 (9.9)	30 (21.1)	0.42	0.19–0.87	**0.033**
Fy^a^Fy^b^|Le^b^|P1(+)|ss	8 (6.6)	22 (15.5)	0.41	0.16–0.96	0.055
RhD(+)|ee|P1(+)|ss	11 (9.1)	30 (21.1)	0.35	0.15–0.74	**0.018**
kk|Le^b^|P1(+)|ss	14 (11.6)	38 (26.8)	0.35	0.17–0.69	**0.016**
Blood type A|ee|P1(+)|ss	7 (5.8)	21 (14.8)	0.30	0.11–0.74	**0.023**
ee|Fy^a^Fy^b^|Jk^a^Jk^b^|ss	7 (5.8)	21 (14.8)	0.35	0.13–0.85	**0.034**
Blood type O|ee|Jk^a^Jk^b^|P1(+)	6 (5.0)	19 (13.4)	0.40	0.14–1.05	0.077
Blood type A|RhD(+)|Cc|P1(+)	8 (6.6)	25 (17.6)	0.29	0.11–0.66	**0.016**
ee|kk|P1(+)|ss	14 (11.6)	40 (28.2)	0.30	0.15–0.60	**0.014**
Blood type A|Cc|Jk^a^Jk^b^|P1(+)	5 (4.1)	17 (12.0)	0.28	0.09–0.75	**0.028**
kk|Jk^a^Jk^b^|P1(+)|ss	9 (7.4)	29 (20.4)	0.33	0.14–0.73	**0.018**
Blood type A|Cc|kk|P1(+)	7 (5.8)	24 (16.9)	0.26	0.10–0.63	**0.016**
Blood type A|Cc|Le^b^|P1(+)	6 (5.0)	21 (14.8)	0.26	0.09–0.64	**0.016**
Jk^a^Jk^b^ |Le^b^|P1(+)|ss	8 (6.6)	27 (19.0)	0.31	0.12–0.72	**0.018**
Blood type A|RhD(+)|P1(+)|ss	6 (5.0)	23 (16.2)	0.25	0.09–0.64	**0.016**
Blood type A|kk|P1(+)|ss	6 (5.0)	23 (16.2)	0.25	0.09–0.63	**0.016**
ee|Jk^a^Jk^b^|P1(+)|ss	7 (5.8)	28 (19.7)	0.24	0.09–0.58	**0.016**
ee|Leb|P1(+)|ss	10 (8.3)	40 (28.2)	0.21	0.09–0.45	**0.003**
ee|Fy^a^Fy^b^|P1(+)|ss	5 (4.1)	23 (16.2)	0.22	0.07–0.59	**0.016**

OR—odds ratio, CI—confidence interval. *p* *—outcome of logistic regression analysis: each row represents the outcome of one logistic regression model with COVID-19 incidence as the dependent variable, the given combination of antigens as the independent variable and gender and age as covariates; the *p* values were adjusted with the FDR method across the analyzed logistic regression models.

**Table 4:** Exploratory nominal correlations of 5-antigen combinations with COVID-19 incidence: comparison between convalescents and controls and adjusted logistic regression analysis.

Combination of Blood Group/Antigens	Convalescents, n (%) (n = 121)	Control Group, n (%) (n = 142)	Adjusted Logistic Regression (Gender and Age)		
			OR	95% CI	*p* *
Combinations positively correlated with COVID-19 incidence (OR > 1)					
Blood type O|kk|Le^b^|P1(+)|MM	13 (10.7)	5 (3.5)	4.44	1.51–15.39	**0.025**
RhD(+)|kk|Le^b^|P1(−)|ss	16 (13.2)	7 (4.9)	2.23	0.89–6.10	0.096
ee|kk |Le^b^|P1(−)|ss	16 (13.2)	7 (4.9)	2.31	0.92–6.32	0.087
Combinations negatively correlated with COVID-19 incidence (OR < 1)					
RhD(+)|kk|Le^b^|P1(+)|ss	11 (9.1)	27 (19.0)	0.44	0.19–0.93	**0.045**
Blood type A|RhD(+)|kk|Le^b^|P1(+)	15 (12.4)	37 (26.1)	0.42	0.20–0.81	**0.025**
ee|Fy^a^Fy^b^|Jk^a^Jk^b^|Le^b^|P1(+)	8 (6.6)	22 (15.5)	0.41	0.16–0.98	0.059
RhD(+)|ee|kk|P1(+)|ss	10 (8.3)	28 (19.7)	0.35	0.15–0.75	**0.025**
RhD(+)|kk|Jk^a^Jk^b^|P1(+)|ss	7 (5.8)	20 (14.1)	0.41	0.15–1.01	0.070
ee|kk|Fy^a^Fy^b^|Jk^a^Jk^b^|ss	7 (5.8)	20 (14.1)	0.37	0.14–0.91	**0.045**
Blood type A|RhD(+)|kk|P1(+)|MM	7 (5.8)	21 (14.8)	0.33	0.12–0.81	**0.029**
Blood type A|RhD(+)|Le^b^|P1(+)|MM	6 (5.0)	19 (13.4)	0.31	0.11–0.79	**0.029**
Blood type A|Cc|ee|kk|P1(+)	6 (5.0)	19 (13.4)	0.29	0.10–0.74	**0.025**
Blood type A|RhD(+)|Cc|Jk^a^Jk^b^|P1(+)	5 (4.1)	17 (12.0)	0.28	0.09–0.75	**0.028**
Blood type A|Cc|kk|Le^b^|P1(+)	6 (5.0)	20 (14.1)	0.27	0.09–0.69	**0.025**
Blood type A|ee|kk|P1(+)|ss	6 (5.0)	20 (14.1)	0.29	0.10–0.73	**0.025**
Blood type A|RhD(+)|Cc|kk|P1(+)	7 (5.8)	24 (16.9)	0.26	0.10–0.63	**0.021**
Blood type A|RhD(+)|Cc|Le^b^|P1(+)	6 (5.0)	21 (14.8)	0.26	0.09–0.64	**0.024**
RhD(+)|ee|Jk^a^Jk^b^|P1(+)|ss	5 (4.1)	18 (12.7)	0.30	0.09–0.83	**0.038**
Blood type A|RhD(+)|ee|P1(+)|ss	5 (4.1)	19 (13.4)	0.25	0.08–0.68	**0.025**
ee|kk|Le^b^|P1(+)|ss	10 (8.3)	36 (25.4)	0.25	0.11–0.52	**0.012**
kk|Jk^a^Jk^b^|Le^b^|P1(+)|ss	7 (5.8)	26 (18.3)	0.28	0.10–0.67	**0.024**
RhD(+)|ee|Le^b^|P1(+)|ss	7 (5.8)	27 (19.0)	0.25	0.09–0.60	**0.018**
ee|kk|Fy^a^Fy^b^|P1(+)|ss	5 (4.1)	20 (14.1)	0.27	0.08–0.73	**0.025**
ee|kk|Jk^a^Jk^b^|P1(+)|ss	6 (5.0)	27 (19.0)	0.22	0.08–0.54	**0.017**
ee|Jk^a^Jk^b^|Le^b^|P1(+)|ss	5 (4.1)	25 (17.6)	0.19	0.06–0.50	**0.017**

OR—odds ratio, CI—confidence interval. *p* *—outcome of logistic regression analysis: each row represents the outcome of one logistic regression model with COVID-19 incidence as the dependent variable, the given combination of antigens as the independent variable and gender and age as covariates; the *p* values were adjusted with the FDR method across the analyzed logistic regression models.

## Data Availability

The datasets used and/or analyzed during the current study are available from the corresponding author upon reasonable request.

## Supplementary Materials

The following supporting information can be downloaded at: https://www.mdpi.com/article/10.3390/life16061038/s1; Table S1: Outcome of logistic regression analysis for COVID-19 incidence with individual antigens as pre-dictors, adjusted with sex and age; Table S2: Outcomes of ROC analysis for COVID-19 incidence, individual antigens; Table S3: Outcomes of ROC analysis of individual antigens for identifying participants without prior COVID-19 incidence, individual antigens; Table S4: Comparison of 3-element antigens combinations between convalescents and control group; Table S5: Bootstrap-based internal validation of logistic regression models evaluating associations between antigen combinations and COVID-19 incidence, for 3-elements combinations; Table S6: Outcomes of ROC analysis for COVID-19 incidence, 3-element combinations; Table S7: Outcomes of ROC analysis of individual antigens for identifying participants without prior COVID-19 incidence, 3-element combinations; Table S8: Comparison of 4-element antigens combinations between convalescents and control group; Table S9: Bootstrap-based internal validation of logistic regression models evaluating associations between antigen combinations and COVID-19 incidence, for 4-elements combinations; Table S10: Outcomes of ROC analysis for COVID-19 incidence, 4-element combinations; Table S11: Outcomes of ROC analysis of individual antigens for identifying participants without prior COVID-19 incidence, 4-element combinations; Table S12: Comparison of 5-element antigens combinations between convalescents and control group; Table S13: Bootstrap-based internal validation of logistic regression models evaluating associations between antigen combinations and COVID-19 incidence, for 5-elements combinations; Table S14: Outcomes of ROC analysis for COVID-19 incidence, 5-element combinations; Table S15: Outcomes of ROC analysis of individual antigens for identifying participants without prior COVID-19 incidence, 5-element combinations.
