# Supplementary material for: Blood Group Antigen Combinations and COVID-19: Complexity, Associations and Possible Clinical Relevance
Source: Life (Basel). 2026 Jun 22;16(6):1038. doi: 10.3390/life16061038 (PMC13300998; doi:10.3390/life16061038)
Supplement: Supplementary file 1 [file life-16-01038-s001.zip › life-4305197-supplementary.pdf]

Supplementary table S1. Outcome of logistic regression analysis for COVID-19 incidence with individual antigens as predictors, adjusted with sex and age.

Variable	Adjusted logistic regression (gender and age)		
	**OR**	**95% CI**	**p***
Blood type; n (%)			
A	0.67	0.40-1.12	0.128
AB	8.44	1.31-167.67	0.058
B	0.78	0.39-1.54	0.483
O	1.46	0.86-2.52	0.166
RhD, n (%)			
presence of D antigen - RhD(+)	1.79	0.90-3.65	0.100
no D antigen - RhD(-)	0.56	0.27-1.11	0.100
Antigen phenotype: C, C^w, c; n (%)			
CC	0.88	0.44-1.72	0.702
Cc	1.19	0.71-2.00	0.506
cc	1.05	0.61-1.82	0.863
CC^w	0.77	0.10-4.36	0.776
C^wc	0.53	0.14-1.74	0.314
Antigen phenotype: E, e; n (%)			
EE	0.54	0.07-2.83	0.484
ee	0.55	0.30-1.02	0.058
Ee	2.08	1.10-4.00	**0.026**
Occurrence of Kell system phenotypes; n (%)			
K [K+k-]	0.00	-	0.987
k [K-k+]	2.06	0.71-6.85	0.201
Kk [K+k]	0.53	0.16-1.59	0.277
Occurrence of Duffy system phenotypes; n (%)			
Fy^a [Fy(a+b-)]	0.96	0.51-1.83	0.912
Fy^b [Fy(a-b+)]	0.98	0.56-1.69	0.929
Fy^aFy^b [Fy(a+b+)]	1.05	0.63-1.74	0.864
Occurrence of Kidd system phenotypes; n (%)			
Jk^a [Jk(a+b-)]	1.26	0.68-2.33	0.458
Jk^b [Jk(a-b-)]	1.00	0.54-1.85	0.999
Jk^aJk^b [Jk(a+b+)]	0.85	0.50-1.42	0.531
Occurrence of Lewis system phenotypes; n (%)			
Le^a [Le(a+b-)]	0.92	0.39-2.16	0.853
Le^b [Le(a-b+)]	0.90	0.47-1.72	0.746
the absence of Le^a and the absence of $Le^{b[}$ [Le(a-b-]	1.33	0.55-3.28	0.526
Occurrence of P1PK system phenotypes; n (%)			
P1(+) [P1]	0.52	0.30-0.90	**0.021**
P1(-) [P2]	1.91	1.11-3.32	**0.021**
Occurrence of MNS system phenotypes:			

Antigen phenotype: M, N; n (%)			
MM	0.83	0.50-1.39	0.488
NN	1.33	0.59-3.03	0.489
MN	1.07	0.64-1.79	0.801
Antigen phenotype: S, s; n (%)			
SS	0.74	0.35-1.53	0.415
ss	0.64	0.38-1.08	0.097
Ss	1.79	1.07-3.02	**0.028**

OR – odds ratio, CI – confidence interval. p - outcome of logistic regression analysis: each row represents outcome of one logistic regression model with COVID-19 incidence as dependent variable, given antigen as independent variable and gender and age as covariates.

Supplementary table S2. Outcomes of ROC analysis for COVID-19 incidence, individual antigens.

**Combination of blood group/antigens**	**AUC (95% CI)**	**Sensitivity**	**Specificity**	**PPV**	**NPV**	**Accuracy**	**p**
Blood type AB	0.525 (0.505;0.550)	0.06	0.99	0.88	0.55	0.56	**0.012**
Blood type 0	0.528 (0.471;0.588)	0.38	0.68	0.50	0.56	0.54	0.341
RhD(+)	0.524 (0.478;0.570)	0.85	0.20	0.47	0.61	0.50	0.301
Cc	0.523 (0.461;0.581)	0.45	0.59	0.49	0.56	0.53	0.452
cc	0.507 (0.451;0.561)	0.33	0.68	0.47	0.54	0.52	0.813
Ee	0.547 (0.499;0.597)	0.26	0.84	0.57	0.57	0.57	0.060
k [K-k+]	0.522 (0.493;0.551)	0.96	0.08	0.47	0.71	0.49	0.148
Fy^aFy^b [Fy(a+b+)]	0.508 (0.450;0.572)	0.50	0.52	0.47	0.55	0.51	0.783
Jk^a [Jk(a+b-)]	0.525 (0.475;0.574)	0.25	0.80	0.52	0.56	0.55	0.323
Jk^b [Jk(a-b-)]	0.506 (0.456;0.555)	0.22	0.79	0.47	0.54	0.53	0.816
the absence of Le^a and the absence of $Le^{b[}$ [Le(a-b-]	0.511 (0.477;0.547)	0.11	0.92	0.52	0.55	0.54	0.528
P1(-)	0.565 (0.506;0.625)	0.40	0.73	0.56	0.59	0.58	**0.026**
NN	0.520 (0.484;0.559)	0.13	0.91	0.55	0.55	0.55	0.295
Ss	0.557 (0.497;0.618)	0.53	0.58	0.52	0.59	0.56	0.066

AUC – area under curve, CI – confidence interval, PPV – positive predictive value, NPV – negative predictive value.

Supplementary table S3. Outcomes of ROC analysis of individual antigens for identifying participants without prior COVID-19 incidence, individual antigens.

**Combination of blood group/antigens**	**AUC (95% CI)**	**Sensitivity**	**Specificity**	**PPV**	**NPV**	**Accuracy**	**p**
Blood type A	0.544 (0.480;0.601)	0.49	0.60	0.59	0.50	0.54	0.153
Blood type B	0.510 (0.464;0.555)	0.18	0.84	0.57	0.47	0.48	0.680
RhD(-)	0.524 (0.480;0.572)	0.20	0.85	0.61	0.47	0.50	0.301
CC	0.509 (0.463;0.553)	0.18	0.83	0.57	0.47	0.48	0.704
CC^w	0.506 (0.488;0.524)	0.03	0.98	0.67	0.46	0.47	0.524
C^wc	0.515 (0.490;0.541)	0.06	0.97	0.69	0.47	0.48	0.251
EE	0.509 (0.491;0.528)	0.04	0.98	0.71	0.46	0.47	0.338
ee	0.538 (0.486;0.589)	0.80	0.27	0.56	0.54	0.56	0.149
K [K+k-]	0.504 (0.500;0.511)	0.01	1.00	1.00	0.46	0.46	0.266
Kk [K+k]	0.518 (0.491;0.545)	0.08	0.96	0.69	0.47	0.48	0.215
Fy^a [Fy(a+b-)]	0.507 (0.461;0.553)	0.20	0.81	0.56	0.46	0.48	0.774
Fy^b [Fy(a-b+)]	0.501 (0.447;0.557)	0.32	0.69	0.54	0.46	0.49	0.960
Jk^aJk^b [Jk(a+b+)]	0.531 (0.471;0.590)	0.59	0.47	0.57	0.50	0.54	0.308
Le^a [Le(a+b-)]	0.503 (0.467;0.540)	0.11	0.90	0.56	0.46	0.47	0.863
Le^b [Le(a-b+)]	0.508 (0.459;0.554)	0.81	0.21	0.55	0.48	0.53	0.738
P1(+)	0.565 (0.506;0.622)	0.73	0.40	0.59	0.56	0.58	**0.026**
MM	0.511 (0.449;0.570)	0.44	0.58	0.55	0.47	0.51	0.718
MN	0.509 (0.449;0.570)	0.46	0.55	0.55	0.47	0.51	0.764
SS	0.512 (0.472;0.552)	0.15	0.88	0.58	0.47	0.48	0.573
ss	0.545 (0.486;0.606)	0.44	0.65	0.60	0.50	0.54	0.138

AUC – area under curve, CI – confidence interval, PPV – positive predictive value, NPV – negative predictive value.

Supplementary table S4. Comparison of 3-element antigens combinations between convalescents and control group.

**Combination of blood group/antigens**	**Convalescents, n (%) (n=121)**	**Control group, n (%) (n=142)**	**p**
cc ǀ Ee ǀ kk	16 (13.2)	7 (4.9)	**0.031**
RhD(+) ǀ cc ǀ Ee	17 (14.0)	8 (5.6)	**0.035**
Cc ǀ kk ǀ P1(-)	22 (18.2)	12 (8.5)	**0.031**
kk ǀ P1(-) ǀ ss	21 (17.4)	12 (8.5)	**0.047**
RhD(+) ǀ kk ǀ P1(-)	40 (33.1)	26 (18.3)	**0.009**
kk ǀ Le^b ǀ P1(-)	40 (33.1)	28 (19.7)	**0.020**
RhD(+) ǀ Le^b ǀ P1(-)	35 (28.9)	25 (17.6)	**0.042**
Blood_A ǀ RhD(+) ǀ kk	36 (29.8)	60 (42.3)	**0.049**
ee ǀ kk ǀ P1(+)	49 (40.5)	77 (54.2)	**0.036**
ee ǀ Le^b ǀ P1(+)	41 (33.9)	68 (47.9)	**0.030**
ee ǀ Jk^aJk^b ǀ P1(+)	25 (20.7)	46 (32.4)	**0.046**
ee ǀ Le^b ǀ ss	26 (21.5)	48 (33.8)	**0.038**
Blood type A ǀ Le^b ǀ P1(+)	22 (18.2)	42 (29.6)	**0.045**
ee ǀ Jk^aJk^b ǀ ss	18 (14.9)	37 (26.1)	**0.038**
Blood type A ǀ kk ǀ P1(+)	25 (20.7)	49 (34.5)	**0.019**
Blood type A ǀ RhD(+) ǀ P1(+)	24 (19.8)	49 (34.5)	**0.012**
ee ǀ Fy^aFy^b ǀ ss	12 (9.9)	28 (19.7)	**0.042**
kk ǀ P1(+) ǀ ss	19 (15.7)	43 (30.3)	**0.009**
Fy^aFy^b ǀ P1(+) ǀ ss	9 (7.4)	24 (16.9)	**0.034**
Jk^aJk^b ǀ P1(+) ǀ ss	11 (9.1)	30 (21.1)	**0.012**
Blood type A ǀ Cc ǀ P1(+)	8 (6.6)	25 (17.6)	**0.013**
Blood type A ǀ P1(+) ǀ ss	8 (6.6)	25 (17.6)	**0.013**
Le^b ǀ P1(+) ǀ ss	15 (12.4)	43 (30.3)	**0.001**
ee ǀ P1(+) ǀ ss	15 (12.4)	44 (31.0)	**0.001**

p - outcome of groups comparison performed with Pearson Chi-square test. p-values are unadjusted (nominal). After false discovery rate (FDR) correction for multiple testing applied to all evaluated antigen combinations within each family, no statistically significant associations were observed.

Supplementary table S5. Bootstrap-based internal validation of logistic regression models evaluating associations between antigen combinations and COVID-19 incidence, for 3-elements combinations.

**Combination of blood group/antigens**	**Adjusted logistic regression (gender and age)**			
	**OR (original)**	**95% CI (original)**	**OR (bootstrap)**	**95% CI (bootstrap)**
**Combinations positively associated with COVID-19 incidence (OR>1)**				
cc ǀ Ee ǀ kk	4.49	1.69-13.36	4.86	1.66-34.35
RhD(+) ǀ cc ǀ Ee	4.15	1.64-11.63	4.37	1.71-16.97
Cc ǀ kk ǀ P1(-)	2.60	1.20-5.91	2.63	1.17-6.08
kk ǀ P1(-) ǀ ss	1.94	0.90-4.34	1.99	0.86-4.67
RhD(+) ǀ kk ǀ P1(-)	2.63	1.44-4.91	2.62	1.43-5.21
kk ǀ Le^b ǀ P1(-)	1.98	1.10-3.58	2.02	1.04-3.75
RhD(+) ǀ Le^b ǀ P1(-)	2.03	1.11-3.79	1.99	1.07-3.95
**Combinations negatively associated with COVID-19 incidence (OR<1)**				
Blood_A ǀ RhD(+) ǀ kk	0.59	0.34-1.00	0.58	0.32-1.06
ee ǀ kk ǀ P1(+)	0.50	0.30-0.85	0.51	0.29-0.85
ee ǀ Le^b ǀ P1(+)	0.52	0.30-0.88	0.51	0.30-0.92
ee ǀ Jk^aJk^b ǀ P1(+)	0.55	0.30-1.00	0.55	0.30-0.92
ee ǀ Le^b ǀ ss	0.47	0.26-0.85	0.47	0.25-0.82
Blood type A ǀ Le^b ǀ P1(+)	0.50	0.27-0.92	0.48	0.25-0.92
ee ǀ Jk^aJk^b ǀ ss	0.48	0.24-0.92	0.48	0.24-0.90
Blood type A ǀ kk ǀ P1(+)	0.47	0.26-0.84	0.46	0.27-0.83
Blood type A ǀ RhD(+) ǀ P1(+)	0.47	0.26-0.84	0.45	0.24-0.89
ee ǀ Fy^aFy^b ǀ ss	0.42	0.19-0.89	0.42	0.18-0.89
kk ǀ P1(+) ǀ ss	0.42	0.22-0.79	0.41	0.21-0.78
Fy^aFy^b ǀ P1(+) ǀ ss	0.42	0.17-0.97	0.41	0.13-1.05
Jk^aJk^b ǀ P1(+) ǀ ss	0.39	0.17-0.84	0.38	0.15-0.84
Blood type A ǀ Cc ǀ P1(+)	0.29	0.11-0.66	0.28	0.10-0.64
Blood type A ǀ P1(+) ǀ ss	0.30	0.12-0.69	0.29	0.10-0.65
Le^b ǀ P1(+) ǀ ss	0.32	0.16-0.62	0.31	0.14-0.64
ee ǀ P1(+) ǀ ss	0.29	0.14-0.56	0.28	0.14-0.53

OR – odds ratio, CI – confidence interval. Each row represents outcome of one logistic regression model with COVID-19 incidence as dependent variable, given combination of antigens as independent variable and gender and age as covariates. Bootstrap columns present the outcomes of internal validation performed using bootstrap resampling (500 replicates). Bootstrap confidence intervals represent percentile-based 95% confidence intervals.

Supplementary table S6. Outcomes of ROC analysis for COVID-19 incidence, 3-element combinations.

Combination of blood group/antigens	AUC (95% CI)	Sensitivity	Specificity	PPV	NPV	Accuracy	p
cc ∣ Ee ∣ kk	0.541 (0.507;0.576)	0.13	0.95	0.70	0.56	0.57	**0.017**
RhD(+) ∣ cc ∣ Ee	0.542 (0.507;0.579)	0.14	0.94	0.68	0.56	0.57	**0.020**
Cc ∣ kk ∣ P1(-)	0.549 (0.507;0.591)	0.18	0.92	0.65	0.57	0.58	**0.019**
kk ∣ P1(-) ∣ ss	0.545 (0.505;0.586)	0.17	0.92	0.64	0.57	0.57	**0.030**
RhD(+) ∣ kk ∣ P1(-)	0.574 (0.524;0.627)	0.33	0.82	0.61	0.59	0.59	**0.006**
kk ∣ Le^b ∣ P1(-)	0.567 (0.512;0.618)	0.33	0.80	0.59	0.58	0.59	**0.014**
RhD(+) ∣ Le^b ∣ P1(-)	0.557 (0.507;0.608)	0.29	0.82	0.58	0.58	0.58	**0.029**

AUC – area under curve, CI – confidence interval, PPV – positive predictive value, NPV – negative predictive value.

Supplementary table S7. Outcomes of ROC analysis of individual antigens for identifying participants without prior COVID-19 incidence, 3-element combinations.

Combination of blood group/antigens	AUC (95% CI)	Sensitivity	Specificity	PPV	NPV	Accuracy	p
Blood type A ∣ RhD(+) ∣ kk	0.563 (0.502;0.619)	0.42	0.70	0.62	0.51	0.55	**0.035**
ee ∣ kk ∣ P1(+)	0.569 (0.510;0.630)	0.54	0.60	0.61	0.53	0.57	**0.026**
ee ∣ Le^b ∣ P1(+)	0.570 (0.512;0.629)	0.48	0.66	0.62	0.52	0.56	**0.021**
ee ∣ Jk^aJk^b ∣ P1(+)	0.559 (0.507;0.611)	0.32	0.79	0.65	0.50	0.54	**0.031**
ee ∣ Le^b ∣ ss	0.562 (0.510;0.616)	0.34	0.79	0.65	0.50	0.54	**0.026**
Blood type A ∣ Le^b ∣ P1(+)	0.557 (0.506;0.611)	0.30	0.82	0.66	0.50	0.54	**0.030**
ee ∣ Jk^aJk^b ∣ ss	0.556 (0.507;0.607)	0.26	0.85	0.67	0.50	0.53	**0.025**
Blood type A ∣ kk ∣ P1(+)	0.569 (0.516;0.622)	0.35	0.79	0.66	0.51	0.55	**0.012**
Blood type A ∣ RhD(+) ∣ P1(+)	0.573 (0.519;0.625)	0.35	0.80	0.67	0.51	0.56	**0.008**
ee ∣ Fy^aFy^b ∣ ss	0.549 (0.507;0.590)	0.20	0.90	0.70	0.49	0.52	**0.025**
kk ∣ P1(+) ∣ ss	0.573 (0.522;0.622)	0.30	0.84	0.69	0.51	0.55	**0.005**
Fy^aFy^b ∣ P1(+) ∣ ss	0.547 (0.507;0.586)	0.17	0.93	0.73	0.49	0.52	**0.018**
Jk^aJk^b ∣ P1(+) ∣ ss	0.560 (0.519;0.603)	0.21	0.91	0.73	0.50	0.53	**0.006**
Blood type A ∣ Cc ∣ P1(+)	0.555 (0.519;0.595)	0.18	0.93	0.76	0.49	0.52	**0.006**
Blood type A ∣ P1(+) ∣ ss	0.555 (0.517;0.594)	0.18	0.93	0.76	0.49	0.52	**0.006**
Le^b ∣ P1(+) ∣ ss	0.589 (0.538;0.636)	0.30	0.88	0.74	0.52	0.57	**<0.001**
ee ∣ P1(+) ∣ ss	0.593 (0.545;0.641)	0.31	0.88	0.75	0.52	0.57	**<0.001**

AUC – area under curve, CI – confidence interval, PPV – positive predictive value, NPV – negative predictive value.

Supplementary table S8. Comparison of 4-element antigens combinations between convalescents and control group.

Combination of blood group/antigens	Convalescents, n (%) (n=121)	Control group, n (%) (n=142)	p
RhD(+) ǀ kk ǀ Fy^b ǀ P1(-)	15 (12.4)	6 (4.2)	**0.027**
RhD(+) ǀ cc ǀ Ee ǀ kk	16 (13.2)	7 (4.9)	**0.031**
kk ǀ Leb ǀ P1(-) ǀ ss	18 (14.9)	8 (5.6)	**0.022**
RhD(+) ǀ kk ǀ P1(-) ǀ ss	18 (14.9)	9 (6.3)	**0.038**
RhD(+) ǀ Cc ǀ kk ǀ P1(-)	21 (17.4)	12 (8.5)	**0.047**
RhD(+) ǀ kk ǀ Le^b ǀ P1(-)	34 (28.1)	22 (15.5)	**0.019**
Blood type A ǀ RhD(+) ǀ kk ǀ Jk^aJk^b	19 (15.7)	38 (26.8)	**0.043**
ee ǀ kk ǀ Jk^aJk^b ǀ ss	17 (14.0)	35 (24.6)	**0.046**
Blood type A ǀ kk ǀ Le^b ǀ P1(+)	19 (15.7)	39 (27.5)	**0.032**
ee ǀ kk ǀ Jk^aJk^b ǀ P1(+)	22 (18.2)	44 (31.0)	**0.025**
Blood type A ǀ RhD(+) ǀ Le^b ǀ P1(+)	18 (14.9)	40 (28.2)	**0.015**
Blood type A ǀ RhD(+) ǀ kk ǀ P1(+)	20 (16.5)	45 (31.7)	**0.007**
RhD(+) ǀ Le^b ǀ P1(+) ǀ ss	12 (9.9)	30 (21.1)	**0.021**
Fy^aFy^b ǀ Le^b ǀ P1(+) ǀ ss	8 (6.6)	22 (15.5)	**0.039**
RhD(+) ǀ ee ǀ P1(+) ǀ ss	11 (9.1)	30 (21.1)	**0.012**
kk ǀ Le^b ǀ P1(+) ǀ ss	14 (11.6)	38 (26.8)	**0.003**
Blood type A ǀ ee ǀ P1(+) ǀ ss	7 (5.8)	21 (14.8)	**0.031**
ee ǀ Fy^aFy^b ǀ Jk^aJk^b ǀ ss	7 (5.8)	21 (14.8)	**0.031**
Blood type O ǀ ee ǀ Jk^aJk^b ǀ P1(+)	6 (5.0)	19 (13.4)	**0.035**
Blood type A ǀ RhD(+) ǀ Cc ǀ P1(+)	8 (6.6)	25 (17.6)	**0.013**
ee ǀ kk ǀ P1(+) ǀ ss	14 (11.6)	40 (28.2)	**0.002**
Blood type A ǀ Cc ǀ Jk^aJk^b ǀ P1(+)	5 (4.1)	17 (12.0)	**0.039**
kk ǀ Jk^aJk^b ǀ P1(+) ǀ ss	9 (7.4)	29 (20.4)	**0.005**
Blood type A ǀ Cc ǀ kk ǀ P1(+)	7 (5.8)	24 (16.9)	**0.009**
Blood type A ǀ Cc ǀ Le^b ǀ P1(+)	6 (5.0)	21 (14.8)	**0.016**
Jk^aJk^b ǀ Le^b ǀ P1(+) ǀ ss	8 (6.6)	27 (19.0)	**0.006**
Blood type A ǀ RhD(+) ǀ P1(+) ǀ ss	6 (5.0)	23 (16.2)	**0.007**
Blood type A ǀ kk ǀ P1(+) ǀ ss	6 (5.0)	23 (16.2)	**0.007**
ee ǀ Jk^aJk^b ǀ P1(+) ǀ ss	7 (5.8)	28 (19.7)	**0.002**
ee ǀ Leb ǀ P1(+) ǀ ss	10 (8.3)	40 (28.2)	**<0.001**
ee ǀ Fy^aFy^b ǀ P1(+) ǀ ss	5 (4.1)	23 (16.2)	**0.003**

p - outcome of groups comparison performed with Pearson Chi-square test. p-values are unadjusted (nominal). After false discovery rate (FDR) correction for multiple testing applied to all evaluated antigen combinations within each family, no statistically significant associations were observed.

Supplementary table S9. Bootstrap-based internal validation of logistic regression models evaluating associations between antigen combinations and COVID-19 incidence, for 4-elements combinations.

<table>
<tr><th rowspan="2">Combination of blood group/antigens</th><th colspan="4">Adjusted logistic regression (gender and age)</th></tr>
<tr><th>OR (original)</th><th>95% CI (original)</th><th>OR (bootstrap)</th><th>95% CI (bootstrap)</th></tr>
<tr><td colspan="5">Combinations positively associated with COVID-19 incidence (OR>1)</td></tr>
<tr><td>RhD(+) | kk | Fy^b | P1(-)</td><td>4.49</td><td>1.61-14.33</td><td>4.52</td><td>1.47-23.28</td></tr>
<tr><td>RhD(+) | cc | Ee | kk</td><td>4.49</td><td>1.69-13.36</td><td>4.52</td><td>1.60-21.21</td></tr>
<tr><td>kk | Leb | P1(-) | ss</td><td>2.23</td><td>0.94-5.74</td><td>2.29</td><td>0.99-7.41</td></tr>
<tr><td>RhD(+) | kk | P1(-) | ss</td><td>2.21</td><td>0.95-5.49</td><td>2.31</td><td>0.93-7.47</td></tr>
<tr><td>RhD(+) | Cc | kk | P1(-)</td><td>2.49</td><td>1.14-5.70</td><td>2.59</td><td>1.11-7.15</td></tr>
<tr><td>RhD(+) | kk | Le^b | P1(-)</td><td>2.29</td><td>1.22-4.38</td><td>2.29</td><td>1.25-4.68</td></tr>
<tr><td colspan="5">Combinations negatively associated with COVID-19 incidence (OR<1)</td></tr>
<tr><td>Blood type A | RhD(+) | kk | Jk^aJk^b</td><td>0.54</td><td>0.28-1.01</td><td>0.53</td><td>0.25-1.01</td></tr>
<tr><td>ee | kk | Jk^aJk^b | ss</td><td>0.49</td><td>0.24-0.94</td><td>0.50</td><td>0.23-0.94</td></tr>
<tr><td>Blood type A | kk | Le^b | P1(+)</td><td>0.47</td><td>0.24-0.89</td><td>0.46</td><td>0.24-0.88</td></tr>
<tr><td>ee | kk | Jk^aJk^b | P1(+)</td><td>0.50</td><td>0.27-0.92</td><td>0.48</td><td>0.28-0.92</td></tr>
<tr><td>Blood type A | RhD(+) | Le^b | P1(+)</td><td>0.45</td><td>0.23-0.86</td><td>0.43</td><td>0.22-0.84</td></tr>
<tr><td>Blood type A | RhD(+) | kk | P1(+)</td><td>0.42</td><td>0.22-0.78</td><td>0.42</td><td>0.21-0.80</td></tr>
<tr><td>RhD(+) | Le^b | P1(+) | ss</td><td>0.42</td><td>0.19-0.87</td><td>0.40</td><td>0.15-0.94</td></tr>
<tr><td>Fy^aFy^b | Le^b | P1(+) | ss</td><td>0.41</td><td>0.16-0.96</td><td>0.41</td><td>0.12-1.00</td></tr>
<tr><td>RhD(+) | ee | P1(+) | ss</td><td>0.35</td><td>0.15-0.74</td><td>0.34</td><td>0.15-0.70</td></tr>
<tr><td>kk | Le^b | P1(+) | ss</td><td>0.35</td><td>0.17-0.69</td><td>0.36</td><td>0.15-0.67</td></tr>
<tr><td>Blood type A | ee | P1(+) | ss</td><td>0.30</td><td>0.11-0.74</td><td>0.29</td><td>0.09-0.72</td></tr>
<tr><td>ee | Fy^aFy^b | Jk^aJk^b | ss</td><td>0.35</td><td>0.13-0.85</td><td>0.35</td><td>0.10-0.88</td></tr>
<tr><td>Blood type O | ee | Jk^aJk^b | P1(+)</td><td>0.40</td><td>0.14-1.05</td><td>0.41</td><td>0.11-1.02</td></tr>
<tr><td>Blood type A | RhD(+) | Cc | P1(+)</td><td>0.29</td><td>0.11-0.66</td><td>0.28</td><td>0.09-0.64</td></tr>
<tr><td>ee | kk | P1(+) | ss</td><td>0.30</td><td>0.15-0.60</td><td>0.30</td><td>0.13-0.61</td></tr>
<tr><td>Blood type A | Cc | Jk^aJk^b | P1(+)</td><td>0.28</td><td>0.09-0.75</td><td>0.27</td><td>0.04-0.74</td></tr>
<tr><td>kk | Jk^aJk^b | P1(+) | ss</td><td>0.33</td><td>0.14-0.73</td><td>0.32</td><td>0.11-0.71</td></tr>
<tr><td>Blood type A | Cc | kk | P1(+)</td><td>0.26</td><td>0.10-0.63</td><td>0.25</td><td>0.09-0.65</td></tr>
<tr><td>Blood type A | Cc | Le^b | P1(+)</td><td>0.26</td><td>0.09-0.64</td><td>0.25</td><td>0.06-0.61</td></tr>
<tr><td>Jk^aJk^b | Le^b | P1(+) | ss</td><td>0.31</td><td>0.12-0.72</td><td>0.31</td><td>0.10-0.70</td></tr>
<tr><td>Blood type A | RhD(+) | P1(+) | ss</td><td>0.25</td><td>0.09-0.64</td><td>0.26</td><td>0.07-0.60</td></tr>
<tr><td>Blood type A | kk | P1(+) | ss</td><td>0.25</td><td>0.09-0.63</td><td>0.25</td><td>0.07-0.61</td></tr>
<tr><td>ee | Jk^aJk^b | P1(+) | ss</td><td>0.24</td><td>0.09-0.58</td><td>0.24</td><td>0.07-0.54</td></tr>
<tr><td>ee | Leb | P1(+) | ss</td><td>0.21</td><td>0.09-0.45</td><td>0.20</td><td>0.07-0.41</td></tr>
<tr><td>ee | Fy^aFy^b | P1(+) | ss</td><td>0.22</td><td>0.07-0.59</td><td>0.22</td><td>0.03-0.58</td></tr>
</table>

OR – odds ratio, CI – confidence interval. Each row represents outcome of one logistic regression model with COVID-19 incidence as dependent variable, given combination of antigens as independent variable and gender and age as covariates. Bootstrap columns present the outcomes of internal validation performed using bootstrap resampling (500 replicates). Bootstrap confidence intervals represent percentile-based 95% confidence intervals.

Supplementary table S10. Outcomes of ROC analysis for COVID-19 incidence, 4-element combinations.

**Combination of blood group/antigens**	**AUC (95% CI)**	**Sensitivity**	**Specificity**	**PPV**	**NPV**	**Accuracy**	**p**
RhD(+) ǀ kk ǀ Fy^b ǀ P1(-)	0.541 (0.507;0.574)	0.12	0.96	0.71	0.56	0.57	**0.014**
RhD(+) ǀ cc ǀ Ee ǀ kk	0.541 (0.507;0.580)	0.13	0.95	0.70	0.56	0.57	**0.017**
kk ǀ Leb ǀ P1(-) ǀ ss	0.546 (0.509;0.583)	0.15	0.94	0.69	0.57	0.58	**0.012**
RhD(+) ǀ kk ǀ P1(-) ǀ ss	0.543 (0.505;0.581)	0.15	0.94	0.67	0.56	0.57	**0.023**
RhD(+) ǀ Cc ǀ kk ǀ P1(-)	0.545 (0.506;0.585)	0.17	0.92	0.64	0.57	0.57	**0.030**
RhD(+) ǀ kk ǀ Le^b ǀ P1(-)	0.563 (0.512;0.612)	0.28	0.85	0.61	0.58	0.59	**0.013**

AUC – area under curve, CI – confidence interval, PPV – positive predictive value, NPV – negative predictive value.

Supplementary table S11. Outcomes of ROC analysis of individual antigens for identifying participants without prior COVID-19 incidence, 4-element combinations.

**Combination of blood group/antigens**	**AUC (95% CI)**	**Sensitivity**	**Specificity**	**PPV**	**NPV**	**Accuracy**	**p**
Blood type A ǀ RhD(+) ǀ kk ǀ Jk^aJk^b	0.555 (0.508;0.603)	0.27	0.84	0.67	0.50	0.53	**0.029**
ee ǀ kk ǀ Jk^aJk^b ǀ ss	0.553 (0.507;0.600)	0.25	0.86	0.67	0.49	0.53	**0.030**
Blood type A ǀ kk ǀ Le^b ǀ P1(+)	0.559 (0.510;0.611)	0.27	0.84	0.67	0.50	0.54	**0.021**
ee ǀ kk ǀ Jk^aJk^b ǀ P1(+)	0.564 (0.510;0.613)	0.31	0.82	0.67	0.50	0.54	**0.016**
Blood type A ǀ RhD(+) ǀ Le^b ǀ P1(+)	0.566 (0.518;0.615)	0.28	0.85	0.69	0.50	0.54	**0.009**
Blood type A ǀ RhD(+) ǀ kk ǀ P1(+)	0.576 (0.523;0.628)	0.32	0.83	0.69	0.51	0.56	**0.004**
RhD(+) ǀ Le^b ǀ P1(+) ǀ ss	0.556 (0.513;0.600)	0.21	0.90	0.71	0.49	0.53	**0.012**
Fy^aFy^b ǀ Le^b ǀ P1(+) ǀ ss	0.544 (0.507;0.581)	0.15	0.93	0.73	0.48	0.51	**0.021**
RhD(+) ǀ ee ǀ P1(+) ǀ ss	0.560 (0.520;0.604)	0.21	0.91	0.73	0.50	0.53	**0.006**
kk ǀ Le^b ǀ P1(+) ǀ ss	0.576 (0.529;0.622)	0.27	0.88	0.73	0.51	0.55	**0.002**
Blood type A ǀ ee ǀ P1(+) ǀ ss	0.545 (0.510;0.581)	0.15	0.94	0.75	0.49	0.51	**0.016**
ee ǀ Fy^aFy^b ǀ Jk^aJk^b ǀ ss	0.545 (0.510;0.579)	0.15	0.94	0.75	0.49	0.51	**0.016**
Blood type O ǀ ee ǀ Jk^aJk^b ǀ P1(+)	0.542 (0.509;0.576)	0.13	0.95	0.76	0.48	0.51	**0.017**
Blood type A ǀ RhD(+) ǀ Cc ǀ P1(+)	0.555 (0.518;0.594)	0.18	0.93	0.76	0.49	0.52	**0.006**
ee ǀ kk ǀ P1(+) ǀ ss	0.583 (0.537;0.631)	0.28	0.88	0.74	0.51	0.56	**0.001**
Blood type A ǀ Cc ǀ Jk^aJk^b ǀ P1(+)	0.539 (0.509;0.572)	0.12	0.96	0.77	0.48	0.51	**0.018**
kk ǀ Jk^aJk^b ǀ P1(+) ǀ ss	0.565 (0.526;0.605)	0.20	0.93	0.76	0.50	0.54	**0.002**
Blood type A ǀ Cc ǀ kk ǀ P1(+)	0.556 (0.519;0.593)	0.17	0.94	0.77	0.49	0.52	**0.004**
Blood type A ǀ Cc ǀ Le^b ǀ P1(+)	0.549 (0.513;0.585)	0.15	0.95	0.78	0.49	0.52	**0.007**
Jk^aJk^b ǀ Le^b ǀ P1(+) ǀ ss	0.562 (0.524;0.602)	0.19	0.93	0.77	0.50	0.53	**0.002**
Blood type A ǀ RhD(+) ǀ P1(+) ǀ ss	0.556 (0.519;0.591)	0.16	0.95	0.79	0.49	0.52	**0.003**
Blood type A ǀ kk ǀ P1(+) ǀ ss	0.556 (0.523;0.593)	0.16	0.95	0.79	0.49	0.52	**0.003**
ee ǀ Jk^aJk^b ǀ P1(+) ǀ ss	0.570 (0.532;0.610)	0.20	0.94	0.80	0.50	0.54	**0.001**
ee ǀ Leb ǀ P1(+) ǀ ss	0.600 (0.554;0.643)	0.28	0.92	0.80	0.52	0.57	**<0.001**
ee ǀ Fy^aFy^b ǀ P1(+) ǀ ss	0.560 (0.526;0.596)	0.16	0.96	0.82	0.49	0.53	**0.001**

AUC – area under curve, CI – confidence interval, PPV – positive predictive value, NPV – negative predictive value.

Supplementary table S12. Comparison of 5-element antigens combinations between convalescents and control group.

Combination of blood group/antigens	Convalescents, n (%) (n=121)	Control group, n (%) (n=142, 54.0%)	p
Blood type 0 ǀ kk ǀ Le^b ǀ P1(+) ǀ MM	13 (10.7)	5 (3.5)	**0.039**
RhD(+) ǀ kk ǀ Le^b ǀ P1(-) ǀ ss	16 (13.2)	7 (4.9)	**0.031**
ee ǀ kk ǀLe^b ǀ P1(-) ǀ ss	16 (13.2)	7 (4.9)	**0.031**
RhD(+) ǀ kk ǀ Le^b ǀ P1(+) ǀ ss	11 (9.1)	27 (19.0)	**0.035**
Blood type A ǀ RhD(+) ǀ kk ǀ Le^b ǀ P1(+)	15 (12.4)	37 (26.1)	**0.009**
ee ǀ Fy^aFy^b ǀ Jk^aJk^b ǀ Le^b ǀ P1(+)	8 (6.6)	22 (15.5)	**0.039**
RhD(+) ǀ ee ǀ kk ǀ P1(+) ǀ ss	10 (8.3)	28 (19.7)	**0.014**
RhD(+) ǀ kk ǀ Jk^aJk^b ǀ P1(+) ǀ ss	7 (5.8)	20 (14.1)	**0.045**
ee ǀ kk ǀ Fy^aFy^b ǀ Jk^aJk^b ǀ ss	7 (5.8)	20 (14.1)	**0.045**
Blood type A ǀ RhD(+) ǀ kk ǀ P1(+) ǀ MM	7 (5.8)	21 (14.8)	**0.031**
Blood type A ǀ RhD(+) ǀ Le^b ǀ P1(+) ǀ MM	6 (5.0)	19 (13.4)	**0.035**
Blood type A ǀ Cc ǀ ee ǀ kk ǀ P1(+)	6 (5.0)	19 (13.4)	**0.035**
Blood type A ǀ RhD(+) ǀ Cc ǀ Jk^aJk^b ǀ P1(+)	5 (4.1)	17 (12.0)	**0.039**
Blood type A ǀ Cc ǀ kk ǀ Le^b ǀ P1(+)	6 (5.0)	20 (14.1)	**0.024**
Blood type A ǀ ee ǀ kk ǀ P1(+) ǀ ss	6 (5.0)	20 (14.1)	**0.024**
Blood type A ǀ RhD(+) ǀ Cc ǀ kk ǀ P1(+)	7 (5.8)	24 (16.9)	**0.009**
Blood type A ǀ RhD(+) ǀ Cc ǀ Le^b ǀ P1(+)	6 (5.0)	21 (14.8)	**0.016**
RhD(+) ǀ ee ǀ Jk^aJk^b ǀ P1(+) ǀ ss	5 (4.1)	18 (12.7)	**0.026**
Blood type A ǀ RhD(+) ǀ ee ǀ P1(+) ǀ ss	5 (4.1)	19 (13.4)	**0.017**
ee ǀ kk ǀ Le^b ǀ P1(+) ǀ ss	10 (8.3)	36 (25.4)	**0.001**
kk ǀ Jk^aJk^b ǀ Le^b ǀ P1(+) ǀ ss	7 (5.8)	26 (18.3)	**0.004**
RhD(+) ǀ ee ǀ Le^b ǀ P1(+) ǀ ss	7 (5.8)	27 (19.0)	**0.003**
ee ǀ kk ǀ Fy^aFy^b ǀ P1(+) ǀ ss	5 (4.1)	20 (14.1)	**0.011**
ee ǀ kk ǀ Jk^aJk^b ǀ P1(+) ǀ ss	6 (5.0)	27 (19.0)	**0.001**
ee ǀ Jk^aJk^b ǀ Le^b ǀ P1(+) ǀ ss	5 (4.1)	25 (17.6)	**0.001**

p - outcome of groups comparison performed with Pearson Chi-square test. p-values are unadjusted (nominal). After false discovery rate (FDR) correction for multiple testing applied to all evaluated antigen combinations within each family, no statistically significant associations were observed.

Supplementary table S13. Bootstrap-based internal validation of logistic regression models evaluating associations between antigen combinations and COVID-19 incidence, for 5-elements combinations.

**Combination of blood group/antigens**	**Adjusted logistic regression (gender and age)**			
	**OR (original)**	**95% CI (original)**	**OR (bootstrap)**	**95% CI (bootstrap)**
**Combinations positively associated with COVID-19 incidence (OR>1)**				
Blood type 0 ǀ kk ǀ Le^b ǀ P1(+) ǀ MM	4.44	1.51-15.39	4.77	1.41-23.84
RhD(+) ǀ kk ǀ Le^b ǀ P1(-) ǀ ss	2.23	0.89-6.10	2.23	0.81-9.02
ee ǀ kk ǀLe^b ǀ P1(-) ǀ ss	2.31	0.92-6.32	2.42	0.97-8.91
**Combinations negatively associated with COVID-19 incidence (OR<1)**				
RhD(+) ǀ kk ǀ Le^b ǀ P1(+) ǀ ss	0.44	0.19-0.93	0.44	0.17-0.93
Blood type A ǀ RhD(+) ǀ kk ǀ Le^b ǀ P1(+)	0.42	0.20-0.81	0.40	0.18-0.85
ee ǀ Fy^aFy^b ǀ Jk^aJk^b ǀ Le^b ǀ P1(+)	0.41	0.16-0.98	0.41	0.12-0.95
RhD(+) ǀ ee ǀ kk ǀ P1(+) ǀ ss	0.35	0.15-0.75	0.34	0.15-0.68
RhD(+) ǀ kk ǀ Jk^aJk^b ǀ P1(+) ǀ ss	0.41	0.15-1.01	0.41	0.13-1.14
ee ǀ kk ǀ Fy^aFy^b ǀ Jk^aJk^b ǀ ss	0.37	0.14-0.91	0.35	0.11-0.83
Blood type A ǀ RhD(+) ǀ kk ǀ P1(+) ǀ MM	0.33	0.12-0.81	0.31	0.09-0.84
Blood type A ǀ RhD(+) ǀ Le^b ǀ P1(+) ǀ MM	0.31	0.11-0.79	0.30	0.10-0.75
Blood type A ǀ Cc ǀ ee ǀ kk ǀ P1(+)	0.29	0.10-0.74	0.29	0.07-0.78
Blood type A ǀ RhD(+) ǀ Cc ǀ Jk^aJk^b ǀ P1(+)	0.28	0.09-0.75	0.26	0.05-0.69
Blood type A ǀ Cc ǀ kk ǀ Le^b ǀ P1(+)	0.27	0.09-0.69	0.27	0.07-0.67
Blood type A ǀ ee ǀ kk ǀ P1(+) ǀ ss	0.29	0.10-0.73	0.28	0.07-0.71
Blood type A ǀ RhD(+) ǀ Cc ǀ kk ǀ P1(+)	0.26	0.10-0.63	0.26	0.06-0.70
Blood type A ǀ RhD(+) ǀ Cc ǀ Le^b ǀ P1(+)	0.26	0.09-0.64	0.25	0.07-0.67
RhD(+) ǀ ee ǀ Jk^aJk^b ǀ P1(+) ǀ ss	0.30	0.09-0.83	0.28	0.05-0.74
Blood type A ǀ RhD(+) ǀ ee ǀ P1(+) ǀ ss	0.25	0.08-0.68	0.24	0.05-0.65
ee ǀ kk ǀ Le^b ǀ P1(+) ǀ ss	0.25	0.11-0.52	0.24	0.09-0.50
kk ǀ Jk^aJk^b ǀ Le^b ǀ P1(+) ǀ ss	0.28	0.10-0.67	0.28	0.07-0.62
RhD(+) ǀ ee ǀ Le^b ǀ P1(+) ǀ ss	0.25	0.09-0.60	0.25	0.06-0.55
ee ǀ kk ǀ Fy^aFy^b ǀ P1(+) ǀ ss	0.27	0.08-0.73	0.28	0.05-0.72
ee ǀ kk ǀ Jk^aJk^b ǀ P1(+) ǀ ss	0.22	0.08-0.54	0.21	0.06-0.52
ee ǀ Jk^aJk^b ǀ Le^b ǀ P1(+) ǀ ss	0.19	0.06-0.50	0.18	0.03-0.45

OR – odds ratio, CI – confidence interval. Each row represents outcome of one logistic regression model with COVID-19 incidence as dependent variable, given combination of antigens as independent variable and gender and age as covariates. Bootstrap columns present the outcomes of internal validation performed using bootstrap resampling (500 replicates). Bootstrap confidence intervals represent percentile-based 95% confidence intervals.

Supplementary table S14. Outcomes of ROC analysis for COVID-19 incidence, 5-element combinations.

**Combination of blood group/antigens**	**AUC (95% CI)**	**Sensitivity**	**Specificity**	**PPV**	**NPV**	**Accuracy**	**p**
Blood type 0 ǀ kk ǀ Le^b ǀ P1(+) ǀ MM	0.536 (0.508;0.568)	0.11	0.96	0.72	0.56	0.57	**0.020**
RhD(+) ǀ kk ǀ Le^b ǀ P1(-) ǀ ss	0.541 (0.507;0.577)	0.13	0.95	0.70	0.56	0.57	**0.017**
ee ǀ kk ǀLe^b ǀ P1(-) ǀ ss	0.541 (0.506;0.575)	0.13	0.95	0.70	0.56	0.57	**0.017**

AUC – area under curve, CI – confidence interval, PPV – positive predictive value, NPV – negative predictive value.

Supplementary table S15. Outcomes of ROC analysis of individual antigens for identifying participants without prior COVID-19 incidence, 5-element combinations.

**Combination of blood group/antigens**	**AUC (95% CI)**	**Sensitivity**	**Specificity**	**PPV**	**NPV**	**Accuracy**	**p**
RhD(+) ǀ kk ǀ Le^b ǀ P1(+) ǀ ss	0.550 (0.509;0.591)	0.19	0.91	0.71	0.49	0.52	**0.020**
Blood type A ǀ RhD(+) ǀ kk ǀ Le^b ǀ P1(+)	0.568 (0.519;0.615)	0.26	0.88	0.71	0.50	0.54	**0.005**
ee ǀ Fy^aFy^b ǀ Jk^aJk^b ǀ Le^b ǀ P1(+)	0.544 (0.510;0.581)	0.15	0.93	0.73	0.48	0.51	**0.021**
RhD(+) ǀ ee ǀ kk ǀ P1(+) ǀ ss	0.557 (0.519;0.597)	0.20	0.92	0.74	0.49	0.53	**0.007**
RhD(+) ǀ kk ǀ Jk^aJk^b ǀ P1(+) ǀ ss	0.541 (0.507;0.577)	0.14	0.94	0.74	0.48	0.51	**0.024**
ee ǀ kk ǀ Fy^aFy^b ǀ Jk^aJk^b ǀ ss	0.541 (0.505;0.579)	0.14	0.94	0.74	0.48	0.51	**0.024**
Blood type A ǀ RhD(+) ǀ kk ǀ P1(+) ǀ MM	0.545 (0.510;0.582)	0.15	0.94	0.75	0.49	0.51	**0.016**
Blood type A ǀ RhD(+) ǀ Le^b ǀ P1(+) ǀ MM	0.542 (0.510;0.576)	0.13	0.95	0.76	0.48	0.51	**0.017**
Blood type A ǀ Cc ǀ ee ǀ kk ǀ P1(+)	0.542 (0.507;0.580)	0.13	0.95	0.76	0.48	0.51	**0.017**
Blood type A ǀ RhD(+) ǀ Cc ǀ Jk^aJk^b ǀ P1(+)	0.539 (0.506;0.570)	0.12	0.96	0.77	0.48	0.51	**0.018**
Blood type A ǀ Cc ǀ kk ǀ Le^b ǀ P1(+)	0.546 (0.513;0.581)	0.14	0.95	0.77	0.49	0.51	**0.011**
Blood type A ǀ ee ǀ kk ǀ P1(+) ǀ ss	0.546 (0.511;0.579)	0.14	0.95	0.77	0.49	0.51	**0.011**
Blood type A ǀ RhD(+) ǀ Cc ǀ kk ǀ P1(+)	0.556 (0.520;0.594)	0.17	0.94	0.77	0.49	0.52	**0.004**
Blood type A ǀ RhD(+) ǀ Cc ǀ Le^b ǀ P1(+)	0.549 (0.515;0.586)	0.15	0.95	0.78	0.49	0.52	**0.007**
RhD(+) ǀ ee ǀ Jk^aJk^b ǀ P1(+) ǀ ss	0.543 (0.511;0.576)	0.13	0.96	0.78	0.48	0.51	**0.011**
Blood type A ǀ RhD(+) ǀ ee ǀ P1(+) ǀ ss	0.546 (0.515;0.581)	0.13	0.96	0.79	0.49	0.51	**0.007**
ee ǀ kk ǀ Le^b ǀ P1(+) ǀ ss	0.585 (0.540;0.626)	0.25	0.92	0.78	0.51	0.56	**<0.001**
kk ǀ Jk^aJk^b ǀ Le^b ǀ P1(+) ǀ ss	0.563 (0.524;0.602)	0.18	0.94	0.79	0.50	0.53	**0.002**
RhD(+) ǀ ee ǀ Le^b ǀ P1(+) ǀ ss	0.566 (0.529;0.604)	0.19	0.94	0.79	0.50	0.54	**0.001**

ee ǀ kk ǀ Fy^aFy^b ǀ P1(+) ǀ ss	0.550 (0.519;0.587)	0.14	0.96	0.80	0.49	0.52	**0.004**
ee ǀ kk ǀ Jk^aJk^b ǀ P1(+) ǀ ss	0.570 (0.533;0.607)	0.19	0.95	0.82	0.50	0.54	**<0.001**
ee ǀ Jk^aJk^b ǀ Le^b ǀ P1(+) ǀ ss	0.567 (0.532;0.605)	0.18	0.96	0.83	0.50	0.54	**<0.001**

AUC – area under curve, CI – confidence interval, PPV – positive predictive value, NPV – negative predictive value.
